# Supplementary material for: The Impact of Footwear on Occupational Task Performance and Musculoskeletal Injury Risk: A Scoping Review to Inform Tactical Footwear
Source: Int J Environ Res Public Health. 2022 Aug 27;19(17):10703. doi: 10.3390/ijerph191710703 (PMC9518076; doi:10.3390/ijerph191710703)
Supplement: Supplementary file 1 [file ijerph-19-10703-s001.zip › Supplementary File S1.pdf]

SUPPLEMENTARY FILE S1: SEARCH STRATEGIES

Database	Search Terms
PubMed	"Boot*"[Title/Abstract] OR "Shoe*"[Title/Abstract] OR "Footwear"[Title/Abstract]) AND ("Occupation*"[Title/Abstract] OR "Profession"[Title/Abstract] OR "Trade*"[Title/Abstract] OR "Job"[Title/Abstract] OR "Work*"[Title/Abstract] OR "Safety"[Title/Abstract] OR "Nurses"[Mesh] OR "Miners"[Mesh] OR "Emergency Responders"[Mesh] OR "Military Personnel"[Mesh] OR "Farmers"[Mesh])
SCOPUS	(Boot*:ti,ab OR Shoe*:ti,ab OR Footwear:ti,ab) AND (Occupation*:ti,ab OR Profession:ti,ab OR Trade*:ti,ab OR Job:ti,ab OR Work*:ti,ab OR Safety:ti,ab OR Nurses/exp OR Miners/exp OR "Emergency Responders"/exp OR "Military Personnel"/exp OR Farmers/exp)
CINAHL	((TI Boot* OR AB Boot*) OR (TI Shoe* OR AB Shoe*) OR (TI Footwear OR A B Footwear)) AND ((TI Occupation* OR AB Occupation*) OR (TI Profession O R AB Profession) OR (TI Trade* OR AB Trade*) OR (TI Job OR AB Job) OR (T I Work* OR AB Work*) OR (TI Safety OR AB Safety) OR (MH Nurses+) OR (MH Miners+) OR (MH "Emergency Responders"+) OR (MH "Military Personnel"+) OR (MH Farmers+))
SportDiscus	((TI "Boot*" OR AB "Boot*") OR (TI "Shoe*" OR AB "Shoe*") OR (TI "Footwe ar" OR AB "Footwear")) AND ((TI "Occupation*" OR AB "Occupation*") OR (TI "Profession" OR AB "Profession") OR (TI "Trade*" OR AB "Trade*") OR (TI "Job" OR AB "Job") OR (TI "Work*" OR AB "Work*") OR (TI "Safety" OR A B "Safety") OR DE "Nurses" OR DE "Miners" OR DE "Emergency Responders" OR DE "Military Personnel" OR DE "Farmers")
Embase	(Boot*:ti,ab OR Shoe*:ti,ab OR Footwear:ti,ab) AND (Occupation*:ti,ab OR Profession:ti,ab OR Trade*:ti,ab OR Job:ti,ab OR Work*:ti,ab OR Safety:ti,ab OR Nurses/exp OR Miners/exp OR "Emergency Responders"/exp OR "Military Personnel"/exp OR Farmers/exp)
Web of Science	(Boot* OR Shoe* OR Footwear) AND (Occupation* OR Profession OR Trade* OR Job OR Work* OR Safety OR Nurses OR Miners OR "Emergency Responders" OR "Military Personnel" OR Farmers)
