# Supplementary material for: The Impact of Footwear on Occupational Task Performance and Musculoskeletal Injury Risk: A Scoping Review to Inform Tactical Footwear
Source: Int J Environ Res Public Health. 2022 Aug 27;19(17):10703. doi: 10.3390/ijerph191710703 (PMC9518076; doi:10.3390/ijerph191710703)
Supplement: Supplementary file 1 [file ijerph-19-10703-s001.zip › Supplementary File S2.pdf]

SUPPLEMENTARY FILE S2: EXCLUDED ARTICLES WITH REASONS

Reason for exclusion	References
Reports not retrieved	[1-13]
Occupational footwear not assessed (includes insoles)	[14-58]
Task performance or musculoskeletal injury risk not assessed	[59-124]
Not occupationally mandated footwear	[125-139]
Not peer-review	[140, 141]
Not published last 20 years	[142-166]
Not translatable	[167]
Not an occupational population	[168-210]
Study protocol	[211]

EXCLUDED REFERENCES:

1. Crowther, M. and M. Gilbertson, The electrician's boot: A primary bone diffuse large B cell lymphoma of the medial cuneiform. Internal Medicine Journal, 2017. **47**: p. 39.
2. Furey, B., Putting Your Best Foot Forward. Firehouse, 2014. **39**: p. A17-A18.
3. Garner, J.C. and C. Wade, Temporal & Spatial Gait Characteristics During Prolonged Exposure to A Normal Surface in Work boots. Conference Proceedings of the Annual Meeting of the American Society of Biomechanics, 2010: p. 609-610.
4. Harris, L., Feet for Life month: workplace footwear. Podiatry Now, 2007. **10**(6): p. 35-38.
5. Harris, L., Occupational podiatry: foot care in the workplace. Podiatry Now, 2012. **15**(5): p. 27-29.
6. Kai Way, L., Slip-Resistance And Abrasion Of New& Used Shoe Soles. Conference Proceedings of the Annual Meeting of the American Society of Biomechanics, 2010: p. 178-179.
7. Laidlaw, L., Comfort drives choice of footwear on 12-hour shift. Nursing Standard, 2008. **22**(26): p. 33-33.
8. Oliver, G.D., et al., Drop Landings In Military Boots. Conference Proceedings Of The Annual Meeting of the American Society of Biomechanics, 2010: p. 876-877.

9. Roberts, D. and R. Nealy, Don't ignore those aching feet. Nursing Update, 2013. **38**(2): p. 6-6.

10. Shematek, G., Spotlight on safety. A question of appropriate footwear. Canadian Journal of Medical Laboratory Science, 2007. **69**(5): p. 215-215.

11. Su, H., L. Song, and J. Zhang, Research on Adaptive Balance Adjustment of Lower Limb Joints and Muscles in the Process of Unexpected Slip. Sheng wu yi xue gong cheng xue za zhi = Journal of biomedical engineering = Shengwu yixue gongchengxue zazhi, 2016. **33**(4): p. 659-665.

12. Ueno, S. and S. Sawada. The effects of walking on dry heat exchange of a newly developed fire-fighter's clothing. in 6th International Thermal Manikin and Modelling Meeting. 2006. Hong Kong, PEOPLES R CHINA.

13. Walbert, H., Who pays for insoles of safety shoes? - It becomes bureaucratic once again. MMW-Fortschritte der Medizin, 2020. **162**(19): p. 40.

14. Alexander, M., If the shoe fits, don't wear it. Journal of Infusion Nursing, 2007. **30**(5): p. 249-250.

15. Alsaffar, N.M.A. and N.H. Neama, Occupational safety and working conditions provided by the municipality of the capital for waste removal workers: Study in one of the municipal departments. Annals of Tropical Medicine and Public Health, 2020. **23**(13B).

16. Andersen, K., et al., Musculoskeletal Lower Limb Injury Risk in Army Populations. Sports Medicine - Open, 2016. **2**(1): p. 1-9.

17. Anderson, J., A.E. Williams, and C. Nester, Development and evaluation of a dual density insole for people standing for long periods of time at work. J Foot Ankle Res, 2020. **13**(1): p. 42.

18. Attwells, R.L., et al., Influence of carrying heavy loads on soldiers' posture, movements and gait. Ergonomics, 2006. **49**(14): p. 1527-37.

19. Baxter, M.L. and D.G. Baxter, Anthropometric characteristics of feet of soldiers in the New Zealand Army. Mil Med, 2011. **176**(4): p. 438-45.

20. Bell, J.L., et al., Evaluation of a comprehensive slip, trip and fall prevention programme for hospital employees. Ergonomics, 2008. **51**(12): p. 1906-25.

21. Brennan, F.H., Jr., et al., Blisters on the battlefield: the prevalence of and factors associated with foot friction blisters during Operation Iraqi Freedom I. Mil Med, 2012. **177**(2): p. 157-62.

22. Caravaggi, P., et al., Plantar pressure analysis of custom-made insoles for safety shoes. Gait & Posture, 2016. **49**: p. S22-S23.

23. Caravaggi, P., et al., In shoe pressure measurements during different motor tasks while wearing safety shoes: The effect of custom made insoles vs. prefabricated and off-the-shelf. Gait Posture, 2016. **50**: p. 232-238.

24. Carballo-Leyenda, B., et al., Fractional contribution of wildland firefighters' personal protective equipment on physiological strain. Frontiers in Physiology, 2018. **9**(AUG).

25. Damalas, C.A. and G. Abdollahzadeh, Farmers' use of personal protective equipment during handling of plant protection products: Determinants of implementation. Sci Total Environ, 2016. **571**: p. 730-6.

26. Ghasemi, M.H. and M. Anbarian, Immediate effects of using insoles with various wedges on center of pressure indices and comfort rating during load lifting. International Journal of Industrial Ergonomics, 2020. **79**.

27. Ghasemi, M.H., M. Anbarian, and H. Esmaeili, Immediate effects of using insoles with various wedges on activation and co-contraction indices of selected trunk muscles during load lifting. Appl Ergon, 2020. **88**: p. 103195.

28. Grier, T.L., et al., Footwear in the United States Army Band: injury incidence and risk factors associated with foot pain. Foot (Edinb), 2011. **21**(2): p. 60-5.

29. Helton, G.L., et al., Association Between Running Shoe Characteristics and Lower Extremity Injuries in United States Military Academy Cadets. Am J Sports Med, 2019. **47**(12): p. 2853-2862.

30. Hesarikia, H., et al., Effect of foot orthoses on ankle and foot injuries in military service recruits: A randomized controlled Trial. Biosciences Biotechnology Research Asia, 2014. **11**(3): p. 1141-1148.

31. Hill, C.M., et al., The Interaction of Cognitive Interference, Standing Surface, and Fatigue on Lower Extremity Muscle Activity. Safety and Health at Work, 2019. **10**(3): p. 321-326.

32. Hirano, T., et al., Effects of foot orthoses on the work of friction of the posterior tibial tendon. Clin Biomech (Bristol, Avon), 2009. **24**(9): p. 776-80.

33. House, C., A. Reece, and D. Roiz de Sa, Shock-absorbing insoles reduce the incidence of lower limb overuse injuries sustained during Royal Marine training. Mil Med, 2013. **178**(6): p. 683-9.

34. Knapik, J.J., Prevention of foot blisters. J Spec Oper Med, 2014. **14**(2): p. 95-7.

35. Kralam, K. and N. Taneepanichskul, Knowledge, Attitude and Practice Towards Personal Protective Equipment Use Among Steel Industry Workers in Thailand. Journal of Health Research, 2016. **30**: p. S161-S165.

36. Kwon, J., et al., Impacts of gender, weather, and workplace differences in farm worker's gear. J Physiol Anthropol, 2015. **34**: p. 39.

37. Landorf, K.B., Foot orthoses can reduce lower limb overuse injury rate. Journal of Physiotherapy, 2011. **57**(3): p. 193.

38. Lavender, S.A., et al., Quantifying the effectiveness of static and dynamic insoles in reducing the tibial shock experienced during walking. Appl Ergon, 2019. **74**: p. 118-123.

39. Leardini, A., et al., Biomechanical evaluation of custom foot-shape based versus off-the-shelf insoles in safety shoes. Foot and Ankle Surgery, 2016. **22**(2): p. 112.

40. Lullini, G., et al., Functional Evaluation of a Shock Absorbing Insole During Military Training in a Group of Soldiers: A Pilot Study. Mil Med, 2020. **185**(5-6): p. e643-e648.

41. Maiwald, C., T.A. Mayer, and T.L. Milani, Alterations of plantar pressure patterns and foot shape after long distance military marching. Footwear Science, 2018. **10**(3): p. 203-213.

42. Maki, B.E., et al., Interventions to promote more effective balance-recovery reactions in industrial settings: new perspectives on footwear and handrails. Ind Health, 2008. **46**(1): p. 40-50.

43. Mattila, V.M., et al., Can orthotic insoles prevent lower limb overuse injuries? A randomized-controlled trial of 228 subjects. Scand J Med Sci Sports, 2011. **21**(6): p. 804-8.

44. Nakano, H., et al., The effect of wearing insoles with a toe-grip bar on occupational leg swelling and lower limb muscle activity: A randomized cross-over study. J Occup Health, 2020. **62**(1): p. e12193.

45. Nealy, R., et al., The Aching Feet of Nurses: An Exploratory Study. MEDSURG Nursing, 2012. **21**(6): p. 354-359.

46. Onyebeke, L.C., et al., Access to properly fitting personal protective equipment for female construction workers. Am J Ind Med, 2016. **59**(11): p. 1032-1040.

47. Pandelani, T., et al., Impact loading response of the MiL-Lx leg fitted with combat boots. International Journal of Impact Engineering, 2016. **92**: p. 26-31.

48. Richardson, A., et al., Interventions to prevent and reduce the impact of musculoskeletal injuries among nurses: A systematic review. Int J Nurs Stud, 2018. **82**: p. 58-67.

49. Shibuya, H., B. Cleal, and P. Kines, Hazard scenarios of truck drivers' occupational accidents on and around trucks during loading and unloading. Accid Anal Prev, 2010. **42**(1): p. 19-29.

50. Speed, G., K. Harris, and T. Keegel, The effect of cushioning materials on musculoskeletal discomfort and fatigue during prolonged standing at work: A systematic review. Appl Ergon, 2018. **70**: p. 300-314.

51. Štajer, T., H. Burger, and G. Vidmar, Influence of casting method on effectiveness of foot orthoses using plantar pressure distribution: a preliminary study. Prosthet Orthot Int, 2011. **35**(4): p. 411-7.

52. Sulong, M.R., et al., Risk factors associated with leptospirosis among town service workers. International Medical Journal, 2011. **18**(2): p. 83-88.

53. Tarrade, T., et al., Are custom-made foot orthoses of any interest on the treatment of foot pain for prolonged standing workers? Appl Ergon, 2019. **80**: p. 130-135.

54. Wardle, S.L. and J.P. Greeves, Mitigating the risk of musculoskeletal injury: A systematic review of the most effective injury prevention strategies for military personnel. J Sci Med Sport, 2017. **20 Suppl 4**: p. S3-s10.

55. Waters, T.R. and R.B. Dick, Evidence of health risks associated with prolonged standing at work and intervention effectiveness. Rehabil Nurs, 2015. **40**(3): p. 148-65.

56. Yates, J. and S. Cahill, What kind of shoes does a social worker wear? A content analysis of four occupational prototypes. British Journal of Guidance & Counselling, 2019. **47**(3): p. 355-370.

57. Yeung, S.S., Do orthotics work as an injury prevention strategy for the military? A systematic review. Physical Therapy Reviews, 2013. **18**(1): p. 49-50.

58. Yoshikawa, T., et al., Needlestick injuries to the feet of Japanese healthcare workers: a culture-specific exposure risk. Infect Control Hosp Epidemiol, 2007. **28**(2): p. 215-8.

59. Acciai, M.C., et al., Plasterer's occupational allergic contact dermatitis from rubber antidegradant polimerized 2,2,4-trimethyl-1,2-dihydroquinoline. Annali Italiani di Dermatologia Allergologica Clinica e Sperimentale, 2006. **60**(3): p. 100-102.

60. Acciai, M.C., et al., Occupational dyshidrotic allergic contact dermatitis from protective gloves and shoes in a worker sensitized to diethyl thiourea and thiurams. Annali Italiani di Dermatologia Allergologica Clinica e Sperimentale, 2007. **61**(1): p. 30-32.

61. Ahc, M., Healthcare Workers at Risk of Slips, Trips, and Falls: Are nonslip shoes part of the answer?...Jennifer L Bell. Hospital Employee Health, 2019. **38**(9).

62. Ahmad, N.A., et al. Perception study on leading factors of slip and fall incidents in manufacturing industry. in 2nd International Materials, Industrial, and Manufacturing Engineering Conference, MIMEC2015. 2015. Bali, INDONESIA.

63. Anderson, J., A.E. Williams, and C. Nester, An explorative qualitative study to determine the footwear needs of workers in standing environments. J Foot Ankle Res, 2017. **10**: p. 41.

64. Bagheri, Z.S., et al., Reducing fall risk for home care workers with slip resistant winter footwear. Appl Ergon, 2021. **90**: p. 103230.

65. Bagheri, Z.S., et al., Slip resistance and wearability of safety footwear used on icy surfaces for outdoor municipal workers. Work, 2019. **62**(1): p. 37-47.

66. Bagheri, Z.S., et al., Selecting slip resistant winter footwear for personal support workers. Work, 2019. **64**(1): p. 135-151.

67. Baumfeld, D., et al., Shoe heel abrasion and its possible biomechanical cause: a transversal study with infantry recruits. J Orthop Surg Res, 2015. **10**: p. 179.

68. Bearman, G., Healthcare personnel (HCP) attire in acute-care, non-surgical settings. International Journal of Infectious Diseases, 2014. **21**: p. 37.

69. Beschorner, K.E., et al., An observational ergonomic tool for assessing the worn condition of slip-resistant shoes. Appl Ergon, 2020. **88**: p. 103140.

70. Bhoyrul, B., et al., A review of non-glove personal protective equipment-related occupational dermatoses reported to EPIDERM between 1993 and 2013. Contact Dermatitis, 2019. **80**(4): p. 217-221.

71. Bracken, T.D., G.S. Sias, and R.M. Patterson, Measured Breakdown Voltage and Leakage Current of Line Worker Boots. Transactions on Power Delivery, 2010. **25**(1): p. 508-517.

72. Chabin, S., M. Toussaint, and Lee. PPE Directive and Live Working footwear. in 11th International Conference on Live Maintenance (ICOLIM). 2014. Budapest, Hungary.

73. Chander, H., J.C. Garner, and C. Wade, Slip outcomes in firefighters: A comparison of rubber and leather boots. Occupational Ergonomics, 2016. **13**(2): p. 67-77.

74. Chen, Z.Y., et al. Footwear slip resistance measurement: The effects of contaminations and sole treads groove width. in 7th Asian International Conference of Leather Science and Technology. 2006. Chengdu, PEOPLES R CHINA: Chinese Leather Industry Assoc.

75. Cho, S.B., H.S. Kim, and S.H. Oh, Green nail syndrome associated with military footwear. Clin Exp Dermatol, 2008. **33**(6): p. 791-3.

76. Cockayne, S., et al., Slip-resistant footwear reduces slips among National Health Service workers in England: A randomised controlled trial. Occupational and Environmental Medicine, 2021.

77. Courtney, T.K., et al., Factors influencing restaurant worker perception of floor slipperiness. J Occup Environ Hyg, 2006. **3**(11): p. 592-8.

78. Courtney, T.K., et al., Perception of slipperiness and prospective risk of slipping at work. Occup Environ Med, 2013. **70**(1): p. 35-40.

79. Courtney, T.K., et al., Factors associated with worker slipping in limited-service restaurants. Inj Prev, 2010. **16**(1): p. 36-41.

80. Courtney, T.K., et al., Slipping as outcome: implications for epidemiologic research on fall-related injuries. Injury Prevention (1353-8047), 2010. **16**: p. A109-A109.

81. da Silva, J.V., et al. A Protective Safety Footwear Device Simulation and Design: An Innovative and Healthy Hybrid Component. in ASME International Mechanical Engineering Congress and Exposition (IMECE2015). 2015. Houston, TX: Amer Soc Mechanical Engineers.

82. Davia-Aracil, M., et al., Design and functionalisation of shoe outsoles with antimicrobial properties using additive manufacturing technologies: industrial applications. Computers in Industry, 2020. **121**: p. 14.

83. Dobson, J.A., et al., The three-dimensional shapes of underground coal miners' feet do not match the internal dimensions of their work boots. Ergonomics, 2018. **61**(4): p. 588-602.

84. Gao, C., I. Holmér, and J. Abeysekera, Slips and falls in a cold climate: underfoot surface, footwear design and worker preferences for preventive measures. Appl Ergon, 2008. **39**(3): p. 385-91.

85. García-Hernández, C., E.J. Sánchez-Álvarez, and J.L. Huertas-Talón, Foot model for tracking temperature of safety boot insoles: application to different insole materials in firefighter boots. Int J Occup Saf Ergon, 2016. **22**(1): p. 12-9.

86. Hoagland, H., Dielectric and electrical hazard shoes. Occup Health Saf, 2011. **80**(4): p. 36-8.

87. Irzmanska, E., Case study of the impact of toecap type on the microclimate in protective footwear. International Journal of Industrial Ergonomics, 2014. **44**(5): p. 706-714.

88. Irzmanska, E. and A. Brochocka, Influence of the Physical and Chemical Properties of Composite Insoles on the Microclimate in Protective Footwear. Fibres & Textiles in Eastern Europe, 2014. **22**(5): p. 89-95.

89. Irzmanska, E. and A. Brochocka, Modified Polymer Materials for Use in Selected Personal Protective Equipment Products. Autex Research Journal, 2017. **17**(1): p. 35-47.

90. Irzmańska, E., M. Charłusz, and R. Irzmański, The use of impedance plethysmography to evaluate the impact of increasing physical activity on blood flow in the lower extremities involving footwear comfort - a preliminary report. Clinical and Experimental Medical Letters, 2011. **52**(1-2): p. 45-51.

91. Irzmanska, E., et al., Evaluation of the Mechanical Parameters of Ultrasonically Welded Textile Composite Structures for Protective Footwear. Fibres & Textiles in Eastern Europe, 2019. **27**(3): p. 99-105.

92. Irzmańska, E. and M. Okrasa, Evaluation of protective footwear fit for older workers (60+): A case study using 3D scanning technique. International Journal of Industrial Ergonomics, 2018. **67**: p. 27-31.

93. Iwanski, J., How to Assess Workers' Needs to Find Proper Protective Footwear. Occupational Health & Safety News, 2021. **90**(1): p. 48-51.

94. Kallis, R., Safe Footwear Improves Worker Outcomes: OSHA- and ASTM-compliant footwear prevents workplace injuries. Occup Health Saf, 2017. **86**(1): p. 8-11.

95. Kelly, R.A., The incidence and prevention of foot problems among male Phase One British Army recruits at an Army Training Regiment. J R Army Med Corps, 2015. **161 Suppl 1**: p. i56-i59.

96. Kim, J.S. A comparison of slip resistance between the grinded outsoles and new ones of fire fighter's shoes. in 8th International Symposium on Safety Science and Technology (ISSST). 2012. Nanjing, China: Elsevier Science Bv.

97. Kuklane, K., et al., Testing cold protection according to EN ISO 20344: is there any professional footwear that does not pass? Ann Occup Hyg, 2009. **53**(1): p. 63-8.

98. Leand, J., Construction Zones. SGB, 2008. **41**(1): p. 33-34.

99. Leggat, P.A. and D.R. Smith, Military training and musculoskeletal disorders. Journal of Musculoskeletal Pain, 2007. **15**(2): p. 25-32.

100. Magalhaes, M.J., et al. Chemical, Biological, Radiological and Nuclear (CBRN) Protective Clothing - A Review. in 11th International Conference on Global Security, Safety, and Sustainability (ICGS3). 2017. Northumbria Univ, London Campus, London, ENGLAND.

101. Maksudova, U., et al. Research of footwear lining materials thermoconductive properties. in 17th World Textile Conference of the Association-of-Universities-for-Textiles (AUTEX) - Shaping the Future of Textiles. 2017. Greece: Iop Publishing Ltd.

102. Mancuso, G., et al., Skin lesions and other foot problems associated with safety footwear. Giornale italiano di medicina del lavoro ed ergonomia, 2017. **38**(4): p. 261-264.

103. Navarro-Triviño, F.J. and R. Ruiz-Villaverde, Chronic foot eczema caused by Bis-GMA from composite work protection boots. Contact Dermatitis, 2020. **82**(3): p. 167-168.

104. Norlander, A., M. Miller, and G. Gard, Perceived risks for slipping and falling at work during wintertime and criteria for a slip-resistant winter shoe among Swedish outdoor workers. Safety Science, 2015. **73**: p. 52-61.

105. Ntatamala, I., Shoe-allergic dermatitis affecting a sanitation worker. Current Allergy and Clinical Immunology, 2018. **31**(3): p. 184-191.

106. Ohata, C. and M. Yoneda, Allergic contact dermatitis due to dazomet absorbed by agricultural rubber boots. Acta Dermato-Venereologica, 2013. **93**(1): p. 81-82.

107. Oñate, E., F. Meyer, and J. Espinoza, On the road toward the development of clothing size standards and safety devices for Chilean workers. Work, 2012. **41 Suppl 1**: p. 5400-2.

108. Oyekale, A.S., Cocoa Farmers' Compliance with Safety Precautions in Spraying Agrochemicals and Use of Personal Protective Equipment (PPE) in Cameroon. Int J Environ Res Public Health, 2018. **15**(2).

109. Park, H., et al., Assessment of Firefighters' needs for personal protective equipment. Fashion and Textiles, 2014. **1**(1).

110. Sapbamrer, R. and A. Thammachai, Factors affecting use of personal protective equipment and pesticide safety practices: A systematic review. Environ Res, 2020. **185**: p. 109444.

111. Siemund, I. and J. Dahlin, Occupational allergic contact dermatitis to dibutyl maleate and dibutyl fumarate present in a safety shoe: A reason for concern? Contact Dermatitis, 2021.

112. Slodownik, D., et al., Textile and Shoe Allergic Contact Dermatitis in Military Personnel. Dermatitis, 2018. **29**(4): p. 196-199.

113. Soares, M.M., et al., Assessment of slip resistance under footwear materials, tread designs, floor contamination, and floor inclination conditions. Work, 2012. **41**: p. 3349-3351.

114. Stolt, M., et al., Nurses' Perceptions of Their Foot Health: Implications for Occupational Health Care. Workplace Health Saf, 2018. **66**(3): p. 136-143.

115. Suo, Q. and D. Zhang, Investigation and identification of factors affecting migrating peasant workers' usage of safety footwear in the Chinese construction industry. Int J Occup Saf Ergon, 2017. **23**(3): p. 424-430.

116. Suo, R.J., et al. Employers' Beliefs to the Usage of Safety Footwear in the Chinese Construction Industry. in International Conference on Computer Information Science and Application Technology (CISAT). 2018. NE Petr Univ, Daqing, PEOPLES R CHINA: Iop Publishing Ltd.

117. Verma, S.K., et al., Workers' experience of slipping in U.S. limited-service restaurants. J Occup Environ Hyg, 2010. **7**(9): p. 491-500.

118. Verma, S.K., et al., A prospective study of floor surface, shoes, floor cleaning and slipping in US limited-service restaurant workers. Occup Environ Med, 2011. **68**(4): p. 279-85.

119. Verma, S.K., et al., Factors associated with use of slip-resistant shoes in US limited-service restaurant workers. Inj Prev, 2012. **18**(3): p. 176-81.

120. Verma, S.K., et al., Transient risk factors for slipping in limited-service restaurants – a case-crossover study. Injury Prevention (1353-8047), 2010. **16**: p. A130-A130.

121. Verma, S.K., et al., Rushing, distraction, walking on contaminated floors and risk of slipping in limited-service restaurants: a case--crossover study. Occup Environ Med, 2011. **68**(8): p. 575-81.

122. Verma, S.K., et al., Duration of slip-resistant shoe usage and the rate of slipping in limited-service restaurants: results from a prospective and crossover study. Ergonomics, 2014. **57**(12): p. 1919-26.

123. Walther, M. and V. Grosse, Forefoot cushioning in safety shoes - A prospective study in the automobile industry. Zentralblatt fur Arbeitsmedizin, Arbeitsschutz und Ergonomie, 2006. **56**(10): p. 312-321.

124. Wang, S., J. Park, and Y. Wang, Cross-cultural comparison of firefighters' perception of mobility and occupational injury risks associated with personal protective equipment. Int J Occup Saf Ergon, 2021. **27**(3): p. 664-672.

125. Borchgrevink, G.E., et al., Does the use of high-heeled shoes lead to fore-foot pathology? A controlled cohort study comprising 197 women. Foot Ankle Surg, 2016. **22**(4): p. 239-243.

126. Chaiklieng, S. and P. Suggaravetsiri, Risk factors for repetitive strain injuries among school teachers in Thailand. Work, 2012. **41 Suppl 1**: p. 2510-5.

127. Chander, H., J.C. Garner, and C. Wade, Heel contact dynamics in alternative footwear during slip events. International Journal of Industrial Ergonomics, 2015. **48**: p. 158-166.

128. Chander, H., et al., Impact of alternative footwear on human balance. Footwear Science, 2016. **8**(3): p. 165-174.

129. Cheol-Ung, K., et al., Wearing Type of Shoes Affects Lower-Limb Muscle Fatigue and Ankle Stability in Young Female Workers. Journal of Exercise Physiology Online, 2020. **23**(2): p. 41-49.

130. Chowning, L.D., J. Krzyszkowski, and J.R. Harry, Maximalist shoes do not alter performance or joint mechanical output during the countermovement jump. J Sports Sci, 2021. **39**(1): p. 108-114.

131. Ghadage, P.P. and J.H. Sagar, Prevalence of neck dysfunction in women using high heeled footwear working in I.T. profession. Indian Journal of Public Health Research and Development, 2020. **11**(5): p. 318-324.

132. Grier, T., et al., Minimalist Running Shoes and Injury Risk Among United States Army Soldiers. Am J Sports Med, 2016. **44**(6): p. 1439-46.

133. Iram, A. and S. Anjum, Low back pain and functional limitations: An outcome of wearing high heels and flat shoes among females. Pakistan Paediatric Journal, 2020. **44**(4 SUPPL): p. 61.

134. Jun, S.P., Y.Y. You, and S.H. Cho, Development of shoes preventing musculoskeletal injuries attached with a ICT convergent smart health care monitoring system. Research Journal of Pharmacy and Technology, 2017. **10**(8): p. 2837-2842.

135. Knapik, J.J., et al., Injury reduction effectiveness of selecting running shoes based on plantar shape. J Strength Cond Res, 2009. **23**(3): p. 685-97.

136. Knapik, J.J., et al., Injury reduction effectiveness of assigning running shoes based on plantar shape in Marine Corps basic training. Am J Sports Med, 2010. **38**(9): p. 1759-67.

137. Knapik, J.J., et al., Injury-reduction effectiveness of prescribing running shoes on the basis of foot arch height: summary of military investigations. J Orthop Sports Phys Ther, 2014. **44**(10): p. 805-12.

138. Sharma, A., et al., To compare the effects of footwear in young female's postural balance in prolonged standing and sitting job. Medico-Legal Update, 2021. **21**(1): p. 77-81.

139. van Kouwenhove, L., et al., Effect of different forefoot rocker radii on lower-limb joint biomechanics in healthy individuals. Gait Posture, 2021. **86**: p. 150-156.

140. Gonda, J., Choosing the Right Safety Shoe for Your Industry: There are several opportunities for injury if a worker does not feel supported by their footwear. Occupational Health & Safety, 2020. **89**(8): p. 32-33.

141. Reilly, M., Selecting proper safety footwear. Occup Health Saf, 2007. **76**(10): p. 72, 74, 76-7 passim.

142. Arndt, A., et al., A comparison of external plantar loading and in vivo local metatarsal deformation wearing two different military boots. Gait Posture, 2003. **18**(2): p. 20-6.

143. Aschan, C., et al., Slip resistance of oil resistant and non-oil resistant footwear outsoles in winter conditions. Safety Science, 2005. **43**(7): p. 373-389.

144. Bentley, T., et al., Investigating slips, trips and falls in the New Zealand dairy farming sector. Ergonomics, 2005. **48**(8): p. 1008-19.

145. Bentley, T.A. and R.A. Haslam, Identification of risk factors and countermeasures for slip, trip and fall accidents during the delivery of mail. Appl Ergon, 2001. **32**(2): p. 127-34.

146. Chaloner, E.J., J. McMaster, and D.E. Hinsley, Principles and problems underlying testing the effectiveness of blast protective footwear. J R Army Med Corps, 2002. **148**(1): p. 38-43.

147. Chang, W.R., C.C. Chang, and S. Matz, Available friction of ladder shoes and slip potential for climbing on a straight ladder. Ergonomics, 2005. **48**(9): p. 1169-82.

148. Chiou, S.S., et al., Effects of environmental and job-task factors on workers' gait characteristics on slippery surfaces. Occupational Ergonomics, 2002. **3**(4): p. 209-223.

149. Dick, T., New boots: weathering the agony of newness. Emerg Med Serv, 2005. **34**(8): p. 60.

150. Dionne, B., Hands on. These boots are made for walkin' & workin'. JEMS: Journal of Emergency Medical Services, 2002. **27**(8): p. 109-110.

151. Dixon, S.J., et al., Biomechanical analysis of running in military boots with new and degraded insoles. Med Sci Sports Exerc, 2003. **35**(3): p. 472-9.

152. Esterman, A. and L. Pilotto, Foot shape and its effect on functioning in Royal Australian Air Force recruits. Part 2: Pilot, randomized, controlled trial of orthotics in recruits with flat feet. Mil Med, 2005. **170**(7): p. 629-33.

153. House, C.M., S.J. Dixon, and A.J. Allsopp, User trial and insulation tests to determine whether shock-absorbing insoles are suitable for use by military recruits during training. Mil Med, 2004. **169**(9): p. 741-6.

154. House, C.M., et al., The influence of simulated wear upon the ability of insoles to reduce peak pressures during running when wearing military boots. Gait Posture, 2002. **16**(3): p. 297-303.

155. Izmerov, N.F., et al., Physiological-occupational requirements with reference to clothes protecting workers from low temperature and methods for the evaluation of heat insulation. Meditsina truda i promyshlennaia ekologiia, 2001(6): p. 27-30.

156. King, P.M., A comparison of the effects of floor mats and shoe in-soles on standing fatigue. Appl Ergon, 2002. **33**(5): p. 477-84.

157. Knapik, J.J., et al., Effect on injuries of assigning shoes based on foot shape in air force basic training. Am J Prev Med, 2010. **38**(1 Suppl): p. S197-211.

158. Leand, J., Work in progress: occupational footwear opportunities expand due to changing demographics and a shift from heavy to light industry. SGB, 2003. **36**(11): p. 40-41.

159. Manning, D.P. and C. Jones, The effect of roughness, floor polish, water, oil and ice on underfoot friction: current safety footwear solings are less slip resistant than microcellular polyurethane. Appl Ergon, 2001. **32**(2): p. 185-96.

160. McPeck, P., Stay on your toes: proper shoes and hosiery can help nurses avoid leg pain. NurseWeek California, 2001. **14**(19): p. 11-12.

161. Milgrom, C., et al., A controlled randomized study of the effect of training with orthoses on the incidence of weight bearing induced back pain among infantry recruits. Spine, 2005. **30**(3): p. 272-275.

162. Mündermann, A., D.J. Stefanyshyn, and B.M. Nigg, Relationship between footwear comfort of shoe inserts and anthropometric and sensory factors. Medicine and Science in Sports and Exercise, 2001. **33**(11): p. 1939-1945.

163. Nagata, H. An analysis of the sliding properties of worker's footwear and clothing on roof surfaces. in Symposium on Metrology of Pedestrian Locomotion and Slip Resistance. 2001. Conshohocken, Pa: American Society Testing and Materials.

164. Rome, K., H.H. Handoll, and R. Ashford, Interventions for preventing and treating stress fractures and stress reactions of bone of the lower limbs in young adults. Cochrane Database Syst Rev, 2005. **2005**(2): p. Cd000450.

165. Schmidt, M.D., S.I. Sulsky, and P.J. Amoroso, Effectiveness of an outside-the-boot ankle brace in reducing parachuting related ankle injuries. Inj Prev, 2005. **11**(3): p. 163-8.

166. Staal, C., et al., Reducing employee slips, trips, and falls during employee-assisted patient activities. Rehabil Nurs, 2004. **29**(6): p. 211-4, 230; discussion 214.

167. Arani, M.D., M. Anbarian, and M.H. Ghasemi, Immediate effects of unstable shoe on myoelectric activity level of selected trunk muscles during load lifting. Scientific Journal of Kurdistan University of Medical Sciences, 2018. **23**(5): p. 121-132.

168. Anderson, J., C. Nester, and A. Williams, Prolonged occupational standing: the impact of time and footwear. Footwear Science, 2018. **10**(3): p. 189-201.

169. Arachchige, S., et al., Muscle Activity during Postural Stability Tasks: Role of Military Footwear and Load Carriage. Safety, 2020. **6**(3): p. 10.

170. Benjamin, D., et al., Comparison of FAP scores with the use of safety footwear and regular walking shoes. Theoretical Issues in Ergonomics Science, 2017. **18**(6): p. 631-642.

171. Buldt, A.K. and H.B. Menz, Incorrectly fitted footwear, foot pain and foot disorders: a systematic search and narrative review of the literature. J Foot Ankle Res, 2018. **11**: p. 43.

172. Chander, H., J.C. Garner, and C. Wade, Impact on balance while walking in occupational footwear. Footwear Science, 2014. **6**(1): p. 59-66.

173. Chander, H., et al., Postural Control in Workplace Safety: Role of Occupational Footwear and Workload. Safety, 2017. **3**(3): p. 8.

174. Chander, H., et al., An analysis of postural control strategies in various types of footwear with varying workloads. Footwear Science, 2021. **13**(2): p. 181-189.

175. Chander, H., et al., Impact of military type footwear and load carrying workload on postural stability. Ergonomics, 2019. **62**(1): p. 103-114.

176. Chander, H., et al., Impact of military type footwear and workload on heel contact dynamics during slip events. International Journal of Industrial Ergonomics, 2018. **66**: p. 18-25.

177. Chander, H., et al., Impact of occupational footwear and workload on postural stability in work safety. Work, 2019. **64**(4): p. 817-824.

178. Chander, H., C. Wade, and J.C. Garner, The influence of occupational footwear on dynamic balance perturbations. Footwear Science, 2015. **7**(2): p. 115-126.

179. Chen, S., J. Jin, and E. Lou. Toward Slip and Fall Prevention: Exploring the Guidance and Challenges of Anti-slip Footwear. in International Symposium on Safety Science and Engineering (ISSSE). 2012. Beijing, PEOPLES R CHINA: Elsevier Science Bv.

180. Chiu, M.C. and M.J. Wang, Professional footwear evaluation for clinical nurses. Appl Ergon, 2007. **38**(2): p. 133-41.

181. Chong, H.C., et al., Knee joint moments during high flexion movements: Timing of peak moments and the effect of safety footwear. Knee, 2017. **24**(2): p. 271-279.

182. DeBusk, H., et al., Influence of military workload and footwear on static and dynamic balance performance. International Journal of Industrial Ergonomics, 2018. **64**: p. 51-58.

183. Dobson, J.A., D.L. Riddiford-Harland, and J.R. Steele, Effects of wearing gumboots and leather lace-up boots on lower limb muscle activity when walking on simulated underground coal mine surfaces. Appl Ergon, 2015. **49**: p. 34-40.

184. Dobson, J.A., D.L. Riddiford-Harland, and J.R. Steele, Effects of wearing gumboots and leather lace-up work boots on plantar loading when walking on a simulated underground coal mine surface. Footwear Science, 2018. **10**(3): p. 139-148.

185. Garner, J.C., et al., Knee Musculature Co-Contraction Following Extended Durations of Walking in Work Boots. Journal of Strength & Conditioning Research, 2011. **25**: p. S74-S75.

186. Geitner, C., et al., Haptic Foot Pedal: Influence of Shoe Type, Age, and Gender on Subjective Pulse Perception. Hum Factors, 2018. **60**(4): p. 496-509.

187. Ghaleb, A.M., et al., Effect of Ambient Oxygen Content, Safety Shoe Type, and Lifting Frequency on Subject's MAWL and Physiological Responses. Int J Environ Res Public Health, 2019. **16**(21).

188. Ghaleb, A.M., et al., Effect of hypoxia, safety shoe type, and lifting frequency on cardiovascular and ventilation responses. International Journal of Industrial Ergonomics, 2020. **80**.

189. Goto, K. and K. Abe, Gait characteristics in women's safety shoes. Appl Ergon, 2017. **65**: p. 163-167.

190. Hill, C.M., et al., Influence of military-type workload and footwear on muscle exertion during static standing. Footwear Science, 2017. **9**(3): p. 169-180.

191. Hill, C.M., et al., Military-Type Workload and Footwear Alter Lower Extremity Muscle Activity during Unilateral Static Balance: Implications for Tactical Athletic Footwear Design. Sports, 2020. **8**(5): p. 10.

192. Jones, T., A. Iraqi, and K. Beschorner, Performance testing of work shoes labelled as slip resistant. Appl Ergon, 2018. **68**: p. 304-312.

193. Jones, T., A. Iraqi, and K. Beschorner, Corrigendum to: Performance testing of work shoes labelled as slip resistant, Appl. Ergon. 68 (2018) 304-312. Applied Ergonomics, 2018. **70**: p. 134-135.

194. Kobayashi, Y., et al., Effect of safety boots with toe spring on foot clearance features during walking. International Journal of Industrial Ergonomics, 2019. **71**: p. 32-36.

195. Krings, B., et al., Impact of occupational footwear during simulated workloads on energy expenditure. Footwear Science, 2018. **10**(3): p. 157-165.

196. Lindsay, C., et al., Shorter work boot shaft height improves ankle range of motion and decreases the oxygen cost of work. Ergonomics, 2021. **64**(4): p. 532-544.

197. Mavor, M.P. and R.B. Graham, The effects of protective footwear on spine control and lifting mechanics. Appl Ergon, 2019. **76**: p. 122-129.

198. Paisis, P., et al., Cypriot and Greek army military boot cushioning: ground reaction forces and subjective responses. Mil Med, 2013. **178**(4): p. e493-7.

199. Phillips, D.B., et al., Influence of work clothing on physiological responses and performance during treadmill exercise and the Wildland Firefighter Pack Test. Appl Ergon, 2018. **68**: p. 313-318.

200. Rawcliffe, A.J., et al., The Effects of British Army Footwear on Ground Reaction Force and Temporal Parameters of British Army Foot Drill. J Strength Cond Res, 2020. **34**(3): p. 754-762.

201. Roshan Fekr, A., et al., Evaluation of Winter Footwear: Comparison of Test Methods to Determine Footwear Slip Resistance on Ice Surfaces. Int J Environ Res Public Health, 2021. **18**(2).

202. Simpson, J.D., et al., The role of military footwear and workload on ground reaction forces during a simulated lateral ankle sprain mechanism. Foot (Edinb), 2018. **34**: p. 53-57.

203. Sinclair, J. and P.J. Taylor, Influence of new military athletic footwear on the kinetics and kinematics of running in relation to army boots. J Strength Cond Res, 2014. **28**(10): p. 2900-8.

204. Sinclair, J., P.J. Taylor, and S. Atkins, Influence of running shoes and cross-trainers on Achilles tendon forces during running compared with military boots. J R Army Med Corps, 2015. **161**(2): p. 140-3.

205. Taylor, N.A., et al., A fractionation of the physiological burden of the personal protective equipment worn by firefighters. Eur J Appl Physiol, 2012. **112**(8): p. 2913-21.

206. Tennant, L., et al., The Effect of Work Boots on Knee Mechanics and the Center of Pressure at the Knee During Static Kneeling. J Appl Biomech, 2015. **31**(5): p. 363-9.

207. Tian, M., et al., Impact of work boots and load carriage on the gait of oil rig workers. Int J Occup Saf Ergon, 2017. **23**(1): p. 118-126.

208. Tian, M., et al., Effects of load carriage and work boots on lower limb kinematics of industrial workers. Int J Occup Saf Ergon, 2018. **24**(4): p. 582-591.

209. Turner, A.J., et al., Impact of Occupational Footwear and Workload on Lower Extremity Muscular Exertion. International Journal of Exercise Science, 2018. **11**(1): p. 331-341.

210. Wade, C. and J.C. Garner, Ankle & Knee Musculature Co-Contraction Following Extended Durations Of Walking In Work boots. Conference Proceedings of the Annual Meeting of the American Society of Biomechanics, 2010: p. 611-612.

211. Cockayne, S., et al., SSHeW study protocol: does slip resistant footwear reduce slips among healthcare workers? A randomised controlled trial. BMJ Open, 2018. **8**(11): p. e026023.
